# Genetic and molecular understanding for the development of methionine-rich maize: a holistic approach

**DOI:** 10.3389/fpls.2023.1249230

**Published:** 2023-09-19

**Authors:** Veena Devi, Bharat Bhushan, Mamta Gupta, Mehak Sethi, Charanjeet Kaur, Alla Singh, Vishal Singh, Ramesh Kumar, Sujay Rakshit, Dharam P. Chaudhary

**Affiliations:** ^1^ Division of Biochemistry, Indian Institute of Maize Research, Ludhiana, Punjab, India; ^2^ Division of Biotechnology, Indian Institute of Maize Research, Ludhiana, Punjab, India; ^3^ Department of Biochemistry, Punjab Agricultural University, Ludhiana, Punjab, India; ^4^ Division of Plant Breeding, Indian Institute of Maize Research, Ludhiana, Punjab, India

**Keywords:** maize, methionine, δ-zein, poultry feed, QTLs, sulphur metabolism

## Abstract

Maize (*Zea mays*) is the most important coarse cereal utilized as a major energy source for animal feed and humans. However, maize grains are deficient in methionine, an essential amino acid required for proper growth and development. Synthetic methionine has been used in animal feed, which is costlier and leads to adverse health effects on end-users. Bio-fortification of maize for methionine is, therefore, the most sustainable and environmental friendly approach. The zein proteins are responsible for methionine deposition in the form of δ-zein, which are major seed storage proteins of maize kernel. The present review summarizes various aspects of methionine including its importance and requirement for different subjects, its role in animal growth and performance, regulation of methionine content in maize and its utilization in human food. This review gives insight into improvement strategies including the selection of natural high-methionine mutants, molecular modulation of maize seed storage proteins and target key enzymes for sulphur metabolism and its flux towards the methionine synthesis, expression of synthetic genes, modifying gene codon and promoters employing genetic engineering approaches to enhance its expression. The compiled information on methionine and essential amino acids linked Quantitative Trait Loci in maize and orthologs cereals will give insight into the hotspot-linked genomic regions across the diverse range of maize germplasm through meta-QTL studies. The detailed information about candidate genes will provide the opportunity to target specific regions for gene editing to enhance methionine content in maize. Overall, this review will be helpful for researchers to design appropriate strategies to develop high-methionine maize.

## 1 Introduction

Maize is a globally important crop and among cereals occupies third place after rice (*Oryza sativa*) and wheat (*Triticum aestivum*). It is also called the “Queen of Cereals” because of its high genetic yield potential among cereals. The worldwide production of maize was around 1123.07 million metric tons (M MT) in 2020-2021 (Food and Agriculture Organization, 2022). The crop has tremendous genetic variability, which enables it to thrive in tropical, subtropical, and temperate climates. The USA, China, Brazil, European Union, Argentina, India, Ukraine, Mexico, South Africa and Russia are the top ten maize-producing countries. In India, maize is grown throughout the year. It is predominantly a *kharif* (rainy season) crop. Total maize production accounts for ~10% of total food grain production in the country. India contributes around 2.80% of maize production with a quantum of 31.51 M MT in 2020-2021 (Food and Agriculture Organization, 2022). Due to much lower water requirements under changing climate with dwindling water resources and higher CO_2_ levels in the atmosphere, maize is going to play the most important role in world agriculture. Along with this, being a C_4_ plant, it has higher photosynthetic efficiency as compared to C_3_ plants.

Maize is consumed as a staple food in Africa, South America and some parts of Asia. In India, about 60% (18.91 M MT) of maize produced is used for livestock and poultry feed, 20% (6.30 M MT) as food, the rest is used as fuel (3.0 M MT) and for industrial purposes (3.2 M MT) (CROPS, 2018). The rapid expansion of the Indian population is changing the consumption patterns, especially of the urban population (Zhou and Staatz, 2016). The increased demand of poultry meat has resulted in the increased production of the feed crops such as maize and soybean (*Glycine max*).

Structurally, the maize kernel consists of an embryo, a much larger endosperm and a pericarp (Wu and Messing, 2014). Nutritionally, maize endosperm contains ~90% starch and 10% protein (Gibbon and Larkins, 2005). Endosperm storage proteins are classified as albumins (3%), globulins (3%), prolamins (also known as zein) (60%) and glutelins (34%). Maize grain protein is nutritionally imbalanced due to deficiency of essential amino acids (methionine [Met], lysine [Lys], tryptophan [Trp] and threonine [Thr]) in the prolamin fraction (Darrigues et al., 2005). As maize is used for feed, so to balance the nutrition in animal feed mixture, corn is usually supplemented with legumes, but Met remains a limiting amino acid in such feed mixtures as all legumes cannot supplement the deficiency of Met (Scott et al., 2004). The level of Met, Lys and Trp is more important than the total protein content in an animal feed mix. So, nutritionally balanced maize is important, considering the fact that the majority of the maize produced is consumed as food and feed.

Plants, unlike animals, synthesize Met *de novo* and are thus a dietary source of this nutrient for animals. However, supplementation of feed with synthetic amino acids and mineral mixture is a common practice in the feed sector. Thus, synthetic amino acids have become a multimillion-dollar industry (Lai and Messing, 2002). The total worldwide Met market was 685–700 million tons (MT) in 2017, which increased by 27% in 2018 (Xiang et al., 2018). Additionally, supplementation of essential amino acids increases the cost of feed, particularly in the case of Trp and Met for which inexpensive supplements are lacking (Darrigues et al., 2005). It was reported that increasing the content of Met in the diet significantly increases the weight of chicks (Panda et al., 2010). So, the maize varieties with enhanced levels of Met along with Trp and Lys, might have great economic potential in developing countries like India, where the feed sector is growing substantially. These Met bio-fortified maize cultivars will be able to replace the use of expensive synthetic Met. Keeping this in view, this article focused on the targeted strategies to enrich the maize germplasm with higher Met content.

### 1.1 Biochemistry and regulation of zein proteins

#### 1.1.1 Biochemistry of zeins

Storage proteins in seeds act as a reservoir which is utilized during early seedling growth. All fractions of maize storage proteins, except prolamins (zeins) are balanced in their amino acid composition. The high proportion of zeins in the endosperm is the primary reason for the poor protein quality of maize (Vasal, 2000) as zeins proteins are deficient in essential amino acids such as Lys and Trp, whereas non-zeins are rich in these amino acids. In maize, among the zein proteins, β- and δ-zein proteins are rich in Met residues. However, these fractions do not accumulate at sufficient levels to balance Met content.

Various mutant maize varieties are available, like *o2* mutant maize lines, as they have high Lys content, but these lines failed to increase the level of Met, rather somewhat decreased its level (Mertz et al., 1964; Phillips and Mcclure, 1985; Wu et al., 2012). According to the maize protein database, 8% of the maize kernel proteins have Met content above 4%, while about 57% of the maize kernel proteins have Lys content above 4% (Wu et al., 2012). Therefore, for the rebalancing of maize seed storage protein composition, allele-specific up-regulation and gene-editing to enhance Met content would be suitable strategies.

Zeins have been categorized based on differences in solubility, molecular weight, ability to form disulfide interactions and sequence of their coding genes (Coleman and Larkins, 1998; Holding and Messing, 2013). The presence of internal tandem variable repeats with blocks of amino acids (mostly proline and glutamine) in all maize zein proteins was shown to be a common characteristic of zein proteins (Geraghty et al., 1981). These zeins have been classified into subfamilies α, β, γ, and δ- Among these four, the β, γ, and δ-zeins have a higher proportion of sulphur-rich amino acids. The δ-zeins are rich in Met (22%), whereas the γ-zeins are abundant in cysteine (Cys); β-zeins have a high percentage of Met and Cys (11%), while α-zeins lack Met and Cys (Wu et al., 2012). The α-zeins (19 and 22 kDa), containing a higher amount of proline and glutamine, account for 50% of total zeins, and are the reason for lower Met, Lys and Trp in maize kernels (Wu et al., 2009). The γ-zeins (50 kDa, 27 kDa and 16 kDa) contain 6.48%, 7.84% and 9.20% Met, respectively. The β- (15 kDa) and δ- (18 and 10 kDa) zeins are relatively Met rich and account for 11%, 27% and 22% of kernel Met, respectively (**Table 1**) (Swarup et al., 1995). The 18 kDa δ-zein have maximum Met among all zeins, because it is formed by the duplication of 10 kDa δ-zein through the allotetraploidization process (Swigonova et al., 2004; Wu et al., 2009). Along with this this δ-zein gene also contains one codon for Lys and two codons for Trp, which is not present in the mature 10 kDa δ-zein gene. Because of this characteristic, 18 kDa δ-zein has a higher nutritional value (Wu et al., 2009).

**Table 1:** Methionine composition in zein protein of different classes (Wu et al., 2012).

Zeins	Molecular weight (kDa)	*Methionine (%)
α	22	0.00
	19 A	0.94
	19 B	0.46
	19 D	0.46
γ	50	1.08
	27	0.49
	16	1.84
β	15	11.25
δ	18	25.26
	10	22.48

*Methionine (%) = % methionine in total seed protein.

#### 1.1.2 General regulation of zein genes

The most abundant α-zeins (19 and 22 kDa) are synthesized by four highly duplicated gene families distributed across six chromosomal locations on 4S consisting of more than 40 genes (Feng et al., 2009). Gene families Z1A, Z1B and Z1D encode the 19 kDa α-zeins, whereas Z1C sub-family encodes 22 kDa α-zeins (Song et al., 2001; Song and Messing, 2002; Feng et al., 2009). On the contrary, the β- (15 kDa), γ- (16, 27 and 50 kDa) and δ- (10 and 18 kDa) zeins are encoded by single-copy genes (Xu and Messing, 2008). The 15 kDa β-zeins are encoded by the *z2β15* gene. The 50, 27 and 16 kDa γ-zeins are encoded by *z2γ50*, *z2γ27* and *z2γ16*, respectively. The 10 kDa and 18 kDa δ-zeins are encoded by *z2δ10* and *z2δ18* genes on chromosomes (ch) 9 and 6, respectively (Xu and Messing, 2008; Liu et al., 2016). Due to their high levels of expression and complexity, zein synthesis serves as a model system to analyze the coordinated genetic regulation of several genes expressed at a specific developmental stage (Soave and Salamini, 1984).

#### 1.1.3 Dzr1: a mutation that increases grain methionine concentration

Expression and accumulation of the 10 kDa δ-zein are regulated post-transcriptionally by the delta zein regulator (*dzr1*) which stabilizes the transcript of *dzs10*. This transcript has 22.5% codons for Met (Kirihara et al., 1988; Cruz-Alvarez et al., 1991; Schickler et al., 1993; Chaudhuri and Messing, 1995; Lai and Messing, 2002). It is also reported that the target sequence of the *dzr1* regulator is located in the untranslated regions (UTRs) of the mRNA of the *dzs10* gene (Lai and Messing, 2002). In maize two different regulation patterns of *dzr1* were reported. One regulatory pattern was derived from the genome and the other was derived from the environment *i.e*. dependent on its parental origin (Chaudhuri and Messing, 1995). In a study conducted by Olsen et al. (2003) when the B101 Met-rich line was crossed with other inbred lines or utilized as a male Met-donor parent, the *dzs10* allele was lost. This suggested that several other genetic factors, apart from *dzr1*, are involved in the expression of *dzs10*. Lai and Messing (2002) developed a Met-enriched transgenic maize line by replacing the cis-acting site (Mo17 allele) of *dzr1* regulation. The removal of the binding site of *dzr1* in *dzs10* uplifts the transcriptional control and resulted in high Met accumulation (Lai and Messing, 2002).

The 18 kDa δ-zein is regulated differently than that of the 10 kDa δ-zein. The UTRs of both the genes are different, to the extent that they regulate their mRNA accumulation through different RNA-protein interactions. The UTRs of 18 kDa δ-zein lack the prolamin box which is present in the 10 kDa δ-zein promoter sequence, which leads to different transcriptional levels in both the zeins (Wu et al., 2009).

### 1.2 Methionine: its source, function and importance in the animal and human sector

#### 1.2.1 Role of dietary methionine in animal systems

Methionine can be procured from both plant- and animal-based products. It is involved in a variety of metabolic functions and plays a vital part in cellular processes. The three most important functions are: 1) trans-methylation to form S-adenosylmethionine (SAM), a primary methyl donor that methylates compounds to form products like creatine, which is used to produce energy in the form of adenosine triphosphate (ATP) during vigorous exercise, and phosphatidylcholine, which is a structural component of cell membranes mostly present on the outer leaflets of membranes and also plays a role in cell signaling; 2) trans-sulphuration to produce Cys, which is then incorporated into glutathione (used to protect against oxidative damage under oxidative stress) or catabolized to taurine (Martinez et al., 2017); and 3) protein synthesis using a pool of Cys, Thr, and Ile derived from protein breakdown. Methionine also has a role in collagen formation, which is a structural component of connective tissues including skin and cartilages. It also aids the liver in metabolizing fat, preventing its storage, as well as protecting arteries from fat accumulation. It acts as a sulphur supplier to the body (Toohey, 2014). Ruan et al. (2017) have extensively reviewed the role of Met in animal immunity. Seymour (2016) have reviewed the role of Met in the diets of transition dairy cows. Methionine has been largely seen in the context of milk protein and fat synthesis as a co-limiting amino acid, however, now it is considered important for supporting the liver function and oxidative balance, besides immunity (Martinez et al., 2017). The dairy cow has increased metabolic and immunological requirements during the stage of calving. Methionine is associated with metabolic balance of proteins, lipids and antioxidants (Pedernera et al., 2010). Cardoso et al. (2021) showed the positive impact of metabolizable protein and rumen-protected Met on the nutritional and immune status of lactating cows. Junior et al. (2021) demonstrated that a correct balance of Met to Lys promotes optimal utilization of amino acids in lactating cows.

Robinson and Bertolo (2016) have reviewed the role of Met in neonates. The authors conclude that Met is highly essential and the only indispensable sulphur amino acid. Methionine availability was being affected by a number of factors, primarily trans-methylation to other molecules. They highlighted the role of dietary methyl donors in ensuring a regular supply of Met. Garlick (2006) reviewed the toxicity of Met in the human body. Normal fluctuations in dietary Met are well tolerated, however, an increase of five times the normal requirement can potentially enhance homocysteine levels, which can increase susceptibility to cardiovascular disease. In infants also, a 2-5 times higher intake can result in impaired growth, however, no long-term consequences are observed. A recent study highlighted the importance of Met metabolism in human longevity (Mota-Martorell et al., 2021). Methionine supplementation enhanced mitochondrial pyruvate uptake and tricarboxylic acid (TCA) cycle activity. The authors implicate enhanced Met trans-sulfuration to be associated with longevity in humans. Disturbances in Met metabolism are also associated with a number of path physiologies. Singhal et al. (2018) implicated decrease in circulating levels of Met in the onset of multiple sclerosis. Siener et al. (2016) demonstrated that L-Met reduces the risk of struvite and calcium phosphate stone in healthy people.

On the other hand, Alachkar et al. (2022) have implicated the role of Met in neuroinflamation. Similarly, Tapia-Rojas et al. (2015) mentioned L-Met as the trigger for Alzheimer’s disease. Pi et al. (2021) have found 5-methylcytosine as an associated factor in high dietary Met-induced Alzheimer’s disease. Similarly, a number of studies indicate the importance of Met restriction as a therapeutic option in cancer biology (Wanders et al., 2020). Yu et al. (2018) have studied short-term Met deprivation as a strategy to reduce body fat, restore normal weight and glycemic control in mice. Navik et al. (2021) have described Met as a double-edged sword considering its varied role in human metabolism. Neubauer and Landecker (2021) have extensively reviewed the life cycle assessment of synthetic Met and have strongly argued for a reconsideration of the public health effects of anthropogenic augmentation of Met in food supply.

Hence, there’s a need to consider the impact of dietary Met on public health in order to frame administrative policies around it. It is also essential to carefully assess the source of Met while measuring its potential effect on health. Plant proteins are taken up by animals, which later enter the food chain. Biological food processing and cooking results in changes to the protein structure and function. Oxidation of the dietary protein has been linked to a number of diseases (Estevez and Luna, 2017). It has also been linked to aging and age-related disorders (Stadtman, 2001). Dominguez et al. (2021) reviewed the issue of protein oxidation in muscle foods. The sulphur containing amino acids, Met and Cys are the most easily oxidizable amino acids. The irreversible oxidative modification of essential amino acids negatively impacts both their bioavailability as well as potential nutritional benefits. He et al. (2018) demonstrated that meat processing results in protein oxidation and aggregation due to changes in surface hydrophobicity and protein secondary structure. Similarly, Soladoye et al. (2015) have reviewed protein oxidation in processed meats. The authors report increase in freed radical generation and decrease of antioxidant activity in cooked meat, both of which contribute to protein oxidation. Methionine is known to be vulnerable to reactive oxygen species (Dorta et al., 2019; Dominguez et al., 2021). Sultana et al. (2009) have demonstrated that Met at position 35 in β-amyeloid protein is highly critical for associated neurotoxicity. This warrants a necessity to study the impact of source of Met and its association in the pathophysiology of Alzheimer’s disease.

It is suggested that along with the bio-fortification, incorporation of antioxidant system in maize will be beneficial for animal feed (Xiong and Guo, 2020). The authors mention that plants contain many antioxidant compounds like tocopherols, polyphenols etc. Several studies suggest that the consumption of antioxidant rich plant food reduce the relative oxidation rate of proteins and lipids in the animal system (Faustman et al., 1998). Although, it is demonstrated that feeding antioxidant-rich plant extracts to chickens improved the oxidative stability of muscle lipids, but not proteins (Smet et al., 2008). Deb-Choudhury et al. (2020) demonstrated that myofibrillar proteins in animal tissue, like myosin, troponin and collagen are highly prone to oxidative protein modifications. Protein oxidation is correlated to the processing severity of food prepration (Estrada, 2019) and need to be taken into account, while accounting for the potential health effects of Met. Recently, He et al. (2022) demonstrated that Met oxidation is responsible for metastasis of pancreatic tumor cells. Given this information, the source of Met (in the form of plant or animal protein) also needs to be carefully considered while re-assessing the Met life cycle as suggested by Neubauer and Landecker (2021). Sato et al. (2021) have demonstrated the potential of amino acid-enriched plant-based therapeutic food for restoring amino acids levels in plasma of malnourished people. With the increasing concern for environmental and health-related issues, it is necessary to make a thorough scientific assessment of the current knowledge to guide administrative policies around human food components.

#### 1.2.2 Ideal sulphur-containing amino acid: methionine or cysteine?

Methionine is an ideal sulphur-containing amino acid, and the reason for choosing Met is that it is an essential amino acid, whereas Cys is not. Another reason for choosing Met over Cys is that Met metabolism solely provides the total sulphur amino acid (TSAA) requirement of the body through the Met trans-sulphuration pathway, as Met acts as a precursor for Cys. Whereas, Cys is not able to act as a precursor for Met because the Met trans-sulphuration pathway is irreversible (Wheeler and Latshaw, 1981; Finkelstein et al., 1988; Finkelstein, 1990; Baker et al., 1996). A number of experiments on Cys as a Met sparing molecule (Wheeler and Latshaw, 1981; Finkelstein et al., 1988; Baker et al., 1996) have been undertaken in which Met was replaced by Cys in the trans-sulphuration pathway and found to be ineffective. The reason behind this is raising Cys and reducing Met in the diet resulted in rising of dietary organic sulphur at the same concentration of total sulphur amino acids. These results suggested that the sparing of Met *via* Cys may alter the quantity of organic sulphur in TSAA (Chung and Baker, 1992; Fatufe, 2004).

### 1.3 Overview of methionine biosynthesis and metabolism

#### 1.3.1 Sulphate reduction and assimilation

**Figure 1:**
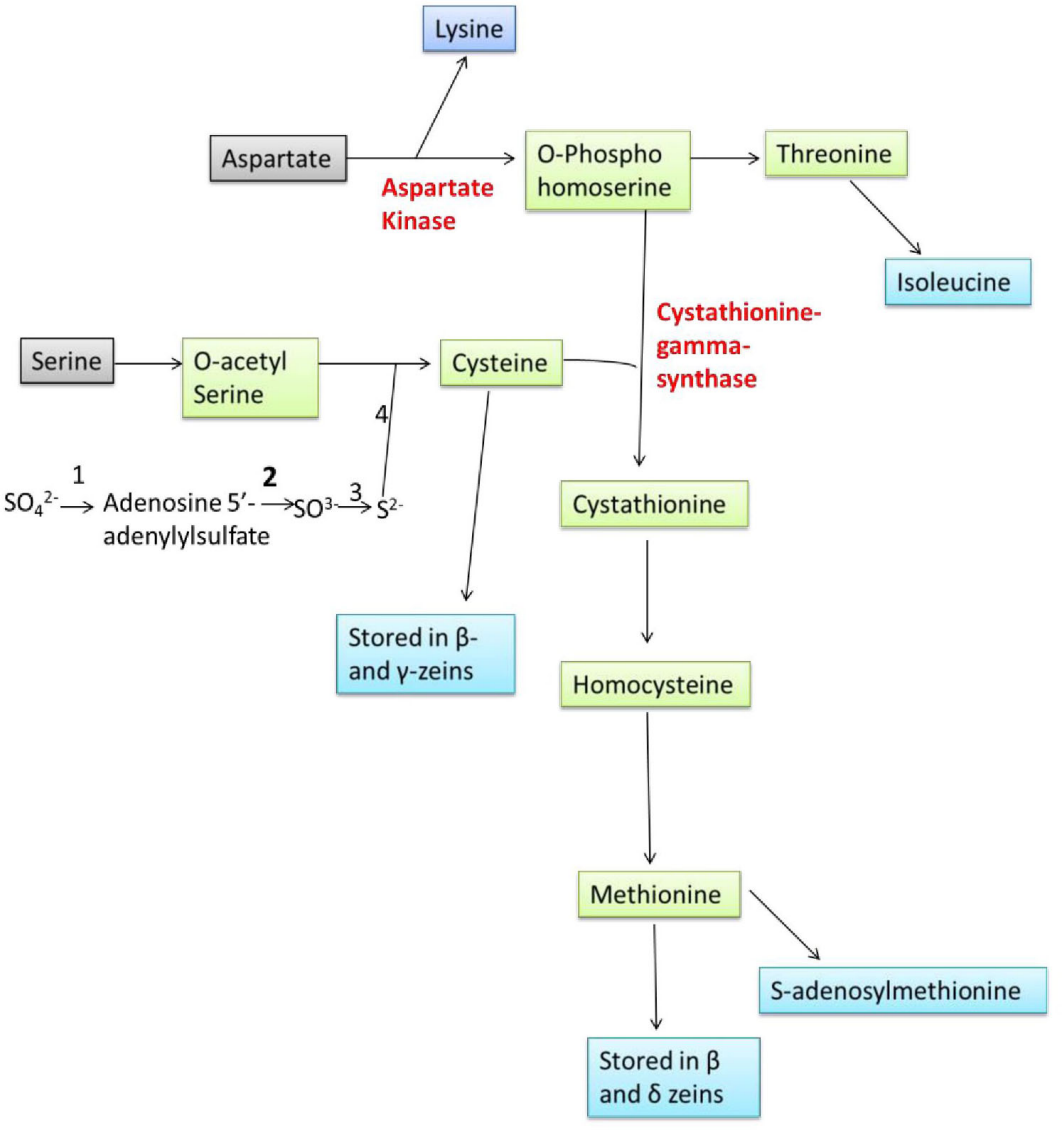
Overview of sulphur assimilation, methionine synthesis and its storage (1: ATP Sulfurylase, 2: APS Reductase, 3: Sulfite Reductase, 4: OAS thiol-lyase).

Amino acids are synthesized in bundle sheath cells during seed development in plants and transferred to the seeds, where they are stored in the form of proteins that are compatible with seed dormancy and germination. Sulphur is taken up by plants from the soil, reduced, and then integrated into Cys and Met in the leaf bundle sheath cells through sulfur assimilation (**Figure 1**). Methionine is generated from Cys as an intermediary step and other molecules including SAM, sulfolipids, and glutathione that also help to reduce the sulphur. Sulfate molecules are reduced to form Cys, and excess of Cys flow for the synthesis of Met. The Cys acts as a donor of thiol moiety to form Met, which indicates that the sulphur uptake and its reduction are the two key steps which are involved in biosynthesis of sulphur containing amino acids in plants (Wu et al., 2012).

Sulfate (SO_4_^2-^) is the stable form of sulphur present in the soil, and plants can takeup this sulfate *via* roots, and convert +6 oxidation state of sulfate to −2 oxidation state of organic sulphur with the help of three enzymes, ATP sulphurylase, adenosine phosphosulfate reductase (APR) and sulfite reductase. The rate of sulfate assimilation is relatively low, accounting for 5% of the rate of nitrate assimilation and 0.1-0.2% of photosynthetic carbon reduction. The activities involved in sulfate assimilation are so minute that it is difficult to elucidate the reaction mechanism involved. The enzymes involved in Cys biosynthesis have been found in the cytosol, plastids and mitochondrion of various plants which reflects the inability of these organelles to transport Cys across their membranes (Wu et al., 2012). SO_4_^2-^ is first converted to adenosine 5’-adenylylsulfate (APS) through the enzyme ATP sulphurylase, APS then is converted to SO_3_^2-^ by the enzyme APR, then to sulfide (S^2-^) via sulfite reductase enzyme. Sulfide (S^2-^) molecule reacts with the O-acetyl serine (OAS) and forming Cys through the enzyme OAS thiol-lyase (OAStL). O-acetyl serine is formed from serine and acetyl CoA through serine acetyltransferase (SAT) enzyme. Formed Cys molecules are used for the synthesis of Cys rich proteins (β- and γ-zeins) and Met, Met then incorporation into the Met-rich δ- zeins protein (**Figure 1**).

#### 1.3.2 Biosynthesis of methionine

**Figure 2:**
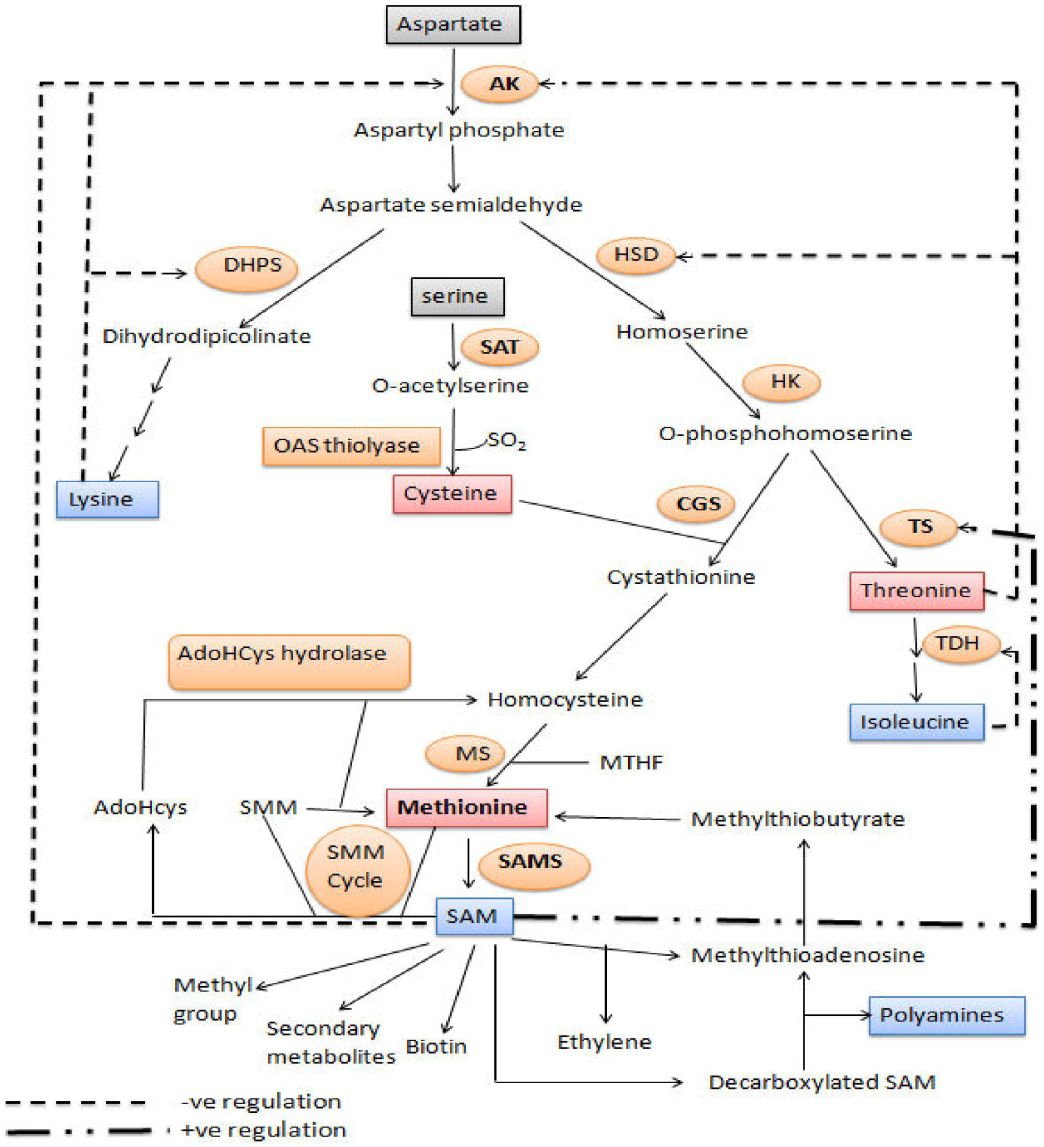
Methionine biosynthesis and metabolism: Diagram of metabolic pathway showing aspartate family pathway, metabolism of methionine and cysteine biosynthesis. Dashed arrows represent feedback inhibition of key enzymes. The bold dashed and dotted arrow represents the stimulation of threonine synthase activity through SAM. (Amir, 2008). DHPS, dihydrodipicolinate synthase; AK, aspartate kinase; HK, homoserine kinase; TS, threonine synthase; HSD, homoserine dehydrogenase; SAT, serine acetyl transferase; TDH, threonine dehydratase; OAS (thio) lyase, O-acetyl serine (thio) lyase; CBL, cystathionine-β-lyase; CGS, cystathionine-γ-synthase; SAM, S-adenosyl methionine; MS, methionine synthase; AdoHcys, adenosylhomocysteine; SAMS, S-adenosyl methionine synthase; SMM, S-methylmethionine; MTHF, methyltetrahydrofolate.

The aspartate family of amino acids consists of four members *viz.*, Met, Thr, Lys and Ile. These amino acids are formed from a common precursor aspartate (**Figure 2**). Aspartate kinase (AK) is the first enzyme that converts aspartate into β-aspartyl semialdehyde. The two amino acids, Thr and Lys accumulation inhibits the AK enzyme through feedback inhibition; thus, their excess quantity regulates Met synthesis. β-aspartyl semialdehyde has two fates, either it goes towards the Lys synthesis or it is converted to O-phosphohomoserine (OPH) *via* homoserine. The OPH either goes towards the synthesis of Thr by the enzyme threonine synthase (TS), or it goes towards the synthesis of Met. Hence, OPH acts as common substrate for both cystathionine-gamma-synthase (CGS) and TS. These two enzymes (TS and CGS) compete with each other to form their respective products (Zeh et al., 2001). Flux of carbon is higher towards the Thr synthesis when Met and SAM contents are higher. SAM content leads to the activation of threonine synthase enzyme. Methionine is formed by three step process from OPH. The Cys and OPH is converted to cystathionine by CGS, cystathionine is converted to homocysteine and then Met using cystathionine-β-lyase (CbL), and methionine synthase (MS), respectively (Hesse and Hoefgen, 2003).

#### 1.3.3 Regeneration of methionine from SAM (SAM Cycle)

Methionine synthesis takes place in the bundle sheath cells of the leaves and accumulates there during the vegetative phase. It is converted to S-methyl-L-methionine (SMM) through the activity of methionine S-methyl transferase and then transported to the seeds in the form of SMM or other appropriate forms during reproductive phase (Bourgis et al., 1999; Bartlem et al., 2000; Kim et al., 2002). The SMM are converted back to the Met in the seeds *via* the enzyme homocysteine S-methyl transferase (HMT) (Ranocha et al., 2001). There are many isoforms of HMT but HMT3 is highly expressive in the seed. The isotope labeling of SMM and Met have shown that SMM produced in rosette leaves is translocated through the stem to the seeds (Cohen et al., 2017). The importance of SMM permeases in the phloem channel (Tan et al., 2010) and role of SMM in maize *mmt* mutants (having insertionally inactivated methionine S-methyl transferase enzyme) is not fully understood because *mmt* mutants and wild type *Arabidopsis thaliana* plants both have the same amount of Met (Kocsis et al., 2003). The transport form of Met at both early (Amir et al., 2019) and late-stage (Gallardo et al., 2007) of seed development is still under investigation in different crops.

It is reported that around 20% of the Met goes towards the synthesis of proteins, while the rest of it is converted to SAM *via* S-adenosylmethionine synthase. Decarboxylated SAM is formed through SAM decarboxylation. It supplies an aminopropyl group during polyamine production, following which Met is reproduced. The SAM is a biologically significant biomolecule because it plays a role in a variety of vital plant processes, including acting as a methyl group donor for DNA methylation and contributing to the discrimination of daughter and parent DNA during DNA repair. It also serves as a secondary metabolite and aids in the production of biotin and ethylene (Amir, 2008).

### 1.4 Genetic understanding of methionine content in maize

#### 1.4.1 Quantitative Trait Loci associated with methionine in maize

In the future, improving Met content in maize kernels will rely on identifying genomic areas and gene networks that are consistently linked to Met production and accumulation. It is necessary to plan experiments on a diverse population with inherent natural variation in order to spot the important genetic areas.

Grain quality is an important feature that regulates the breeding programmes. The essential genes regulating the composition and structure of endosperm have been studied extensively. Multiple modifier genes contribute to superior grain quality under different genetic backgrounds (Ciceri et al., 2000; Huang et al., 2004). The marker-assisted breeding strategy has great potential to enhance the efficiency of the selection of desired traits (Babu et al., 2005). Quality Protein Maize (QPM) breeding programmes can be accelerated by using markers associated with essential amino acid, *opaque2 (o2)* and kernel hardness. Several studies were directed to identify QTLs associated with endosperm modification and protein quality including Met levels. Rojas et al. (2010) screened parental lines for differences in Met content; along with it, they identified four QTLs associated with Met content which together explained 57.3% variation among genotypes. Essential alleles that enhanced Met content were contributed by B73O2 inbred line. Among four reported QTLs, two QTLs were located near QTLs for Met, Lys and Trp-related traits. A QTL cluster on chromosome Ch7L is essentially associated with genes regulating Met and Trp synthesis. Along with it, a QTL cluster on Ch8S contains QTLs for three essential amino acids (Met, Trp, Lys). Both QTL clusters showed 73.9% phenotypic variance for the level of these three amino acids indicating the importance of clusters in the overall protein content of maize. Breeders are more interested in genes and QTLs that are directly involved in amino acid synthesis (metabolic pathways), such as four QTLs governing Met accumulation that were linked to genes regulating Met synthase (Lawrence et al., 2007). Similarly, the anthranilate synthase gene was located with the QTLs involved in phenotypic variation for Met, Trp and Lys accumulation (Lawrence et al., 2007).

#### 1.4.2 Additional genetic components that regulate the methionine levels in maize

Kernel texture is a key feature in bio-fortified maize, along with essential amino acid synthesis in a balanced way. The hard kernel texture is negatively correlated with Lys content. It is established that endosperm modification in *o2* lines decreases the Lys accumulation in contrast to unmodified counterparts. This decrease in Lys content in modified kernels is justified by increased zein content (Robutti et al., 1974).

Additionally, the Met and Trp accumulation also show an inverse relationship. In *o2* genotypes with modified endosperm, Trp content was higher than Met as compared to normal maize inbred (Bantte and Prasanna, 2004; Scott et al., 2004). Amino acids Lys and Met are synthesized *via* a similar metabolic route, with aspartate (Asp) as a common precursor (Azevedo et al., 2006), whereas the chorismate pathway is involved in Trp production which is also crucial for secondary metabolite production (Radwanski and Last, 1995). Although numerous research has been conducted to better understand metabolic processes for amino acid synthesis, very little is known about the genetic regulation of these metabolic pathways in maize (Azevedo et al., 2006). Free amino acids (FAA) are another important nutrition quality trait and their QTLs are reported on Ch2L, Ch2S, Ch3S and Ch7L. Later 11 QTLs for free amino acid content were reported on chromosome 10 (Wang and Larkins, 2001). A gene for AK (*Ask2*) was found to be linked to a QTL on Ch2L affecting FAA content (Wang et al., 2007). In another study, among six reported QTLs for Met content, two QTLs on chromosome 2 were essentially associated with genes regulating Met content in maize. The rest were located as one QTL in Ch3, two QTLs in Ch4 and one in Ch8. The four QTLs associated with Met/ATT (aspartate-derived amino acid family related traits) were located on Ch1, 2 and 8, whereas QTLs for Met/Total trait were clustered on Ch1, 2, 3, 6 and 7 (Deng et al., 2017).

In multiple studies, several QTLs on different chromosomes are mapped for increasing amino acid content in the endosperm. The QTL cluster identified for Trp and Met was mapped close to the *o2* gene; however, although *o2* should not be segregated in tested population, it is unlikely to be associated with QTLs, which leaves AK and a 27 kDa γ-zein gene to be potential candidates found in this region (Robutti et al., 1974; Azevedo et al., 2006; Lawrence et al., 2007; Rojas et al., 2010).

#### 1.4.3 QTLs associated with methionine in orthologous cereal crop

Rice, like maize, is the focus of multiple studies aimed at increasing Met content and its linked genetic regions. Research published in 2002 found 80 QTLs for 19 amino acid content in milled rice, with QTLs for Met content accounting for over 7.4% of the variation (Wang et al., 2008). The 48 and 64 QTLs and 12 QTL clusters for amino acid accumulation were elucidated by Zhong et al. (2011) including qAa1 on Ch1, qAa7 on Ch7 and qAA9 on Ch9. Among these clusters, qAa1 and qAa9 negatively and qAa7 positively regulated the amino acid accumulation. Thus, the QTLs specifically affecting Met content were located on ch1, 9 and 7. In another study, a total of 17 QTLs for amino acid content were reported on six chromosomes *viz.* 1, 3, 6, 7, 8 and 10. Five of the 17 QTLs, qAAC6.1, qAAC6.2, qAAC7.1, qAAC7.2, and qAAC8.2, were shown to be linked to important amino acid genes. Certain QTLs (qAAC6.1 and qAAC7.1) from the reported study had multiple effects on more than one amino acid. Biosynthesis of almost 11 amino acids (Alanine (Ala), Arginine (Arg), Aspartate (Asp), Glutamic acid (Glx), Glycine (Gly), Isoleucine (Ile), Leucine (Leu), Met, Phenylalanine (Phe), Tyrosine (Tyr), and Valine (Val)) was contributed by QTL qAAC6.1 (Jang et al., 2020).

According to the compilation of various studies in rice, it can be concluded that QTLs majorly affecting amino acid accumulation are located on eight chromosomes *viz*. 1, 2, 3, 4, 6, 7, 8, 10. A total of 5 QTL clusters on chromosomes 1, 2, 7 and 8 which majorly affect amino acid accumulation, specifically the region of 1.5-5.19 Mb on Ch1 that has 4 QTLs for amino acid content (Wang et al., 2007; Zhong et al., 2011; Yoo, 2017; Jang et al., 2020). The genetic variations are controlled by QTLs, but none of these have yet been cloned (Demidov et al., 2003). Therefore, the identification of relevant QTLs and their effect on the Met content may lead to developing new maize inbreds with higher Met storage proteins which can be utilized to target specific genomic regions that have been conserved over time for amino acid accumulation and protein quality attributes. The studies on QTLs associated with Met in maize and rice crops are listed in **Table 2**.

**Table 2:** List of detail of QTLs linked with methionine, essential amino acids, and total amino acid content in maize and rice.

Traits	QTL name	Chr.	Marker/interval (Mbp)	LOD value	R ^2^	References
Maize						
Methionine	*qMet5*	5	bnlg1046	3.76	8.9	Rojas et al., 2010
	*qMet7*	7	umc2142	8.91	20.7	
	*qMet8*	8	umc1304	5.07	18.3	
Methionine	*qMet2*	2	PZE-102080745	4.09	8.84	Deng et al., 2017
	*qMet2.1*	2	SYN1715	3.78	7.88	
	*qMet3*	3	PZE-103099938	4.38	8.93	
	*qMet4*	4	SYN2317	3.53	8.49	
	*qMet4.1*	4	PZE-104138961	4.15	8.70	
	*qMet8*	8	PZE-108087230	3.76	8.95	
Methionine/ATT	*qMet/ATT1*	1	ZM013506-0433	4.38	10.33	
	*qMet/ATT2*	2	PZE-102080745	4.97	10.65	
	*qMet/ATT8*	8	PZE-108087230	3.32	7.69	
Methionine/Total amino acids	*qMet/Total1*	1	ZM013506-0433	3.02	6.52	
	*qMet/Total2*	2	PZE-102080745	5.01	10.86	
	*qMet/Total3*	3	SYN37729	4.63	9.09	
	*qMet/Total3.1*	3	PZE-103075963	4.09	7.96	
	*qMet/Total3.2*	3	PUT-163a-101384606-6	3.78	7.59	
	*qMet/Total6*	6	PZE-106006541	3.57	8.49	
	*qMet/Total7*	7	SYNGENTA3646	3.42	7.29	
Free amino acids	*qFAA*	2L	bmc1633- bmc1329	—–	11	Wang and Larkins, 2001
	*qFAA*	2S	bmc1537-bmc2248	—–	10	
	*qFAA*	3S	bmc1904-bmc2136- bmc1452	—–	15	
	*qFAA*	7L	bmc2328b- phi045	—–	10	
Rice						
Ala/Arg/Asx/Glx/Gly/Ile/Leu /Met/Phe/Tyr/Val	*qAAC6.1*	6	3.59	16.9	10.81	Jang et al., 2020
Methionine	*qAAC6.2*	6	4.21	9.3	5.23	
Total amino acid	*qAa1*	1	RM493-RM562 (12.3-14.6)	12.3	24.2	Zhong et al., 2011
	*qAa9*	9	RM328-RM107 (19.7-20.1)	8.1	13.2	
Eaa/Total/Asp/Thr/Glu/Gly/Ala/Cys/Tyr/Pro	*qAA.1*	1	RM472-RM104 (37.9-40.2)	—–	—–	Wang et al., 2007
Pro/Gly/Met/Arg	*qAA.7*	7	RM125-RM214 (5.5-12.8)	—–	—–	

The present review’s compiled information on QTL can be employed in meta-QTL analysis to investigate the congruency of the identified regions related to certain traits of interest. Regions associated in rice could be used for identifying hotspot genomic regions in maize. The information from the compiled meta-QTL studies will be used for the development of trait-specific KASP (kompetitive allele-specific PCR) markers which will be an important stepping stone to accelerating the maize nutritional quality program globally.

### 1.5 Dissection and understanding of proteome rebalance to enhance methionine level

The proportion of essential amino acids (Met, Lys) in maize kernels is determined by the type of maize protein. In previous investigations, high δ-zein lines were found to be sufficient to substitute synthetic Met in a complete feed for poultry and human consumption (Messing and Fisher, 1991). However, for the development of maize lines having a higher content of both Lys and Met, a dissection of the mechanism underlying proteome rebalancing is required. Accumulation of Met-rich δ-zein was associated with the post-transcriptional regulation of its mRNA (Schickler et al., 1993; Lai and Messing, 2002), whereas high-Lys maize lines (*o2* mutant) depend on the compensatory increase of Lys-rich non-zeins. According to the mechanism, an increase in Lys level tends to be accompanied by a decrease in Met content (Mertz et al., 1964; Phillips and Mcclure, 1985; Wu et al., 2012). The decreased Met level in Lys-rich *o2* mutant is related to *o2* mediated reduction of β- and δ-zeins. The proteomic analysis also reports that only 8% of proteins in maize kernel possess Met above 4%, whereas, 57% of proteins have Lys above 4% (Wu et al., 2012). As a result, the mechanisms for accumulating these two amino acids differ throughout seed development, and both must be targeted simultaneously in order to develop nutritionally superior maize lines. Another problem with targeting Met-rich lines is its linkage with a reduced level of Cys-rich γ-zein (27 kDa) (Newell et al., 2014). These studies suggest that increased content of Met during seed storage needs higher reduced sulphur flow towards Met synthesis from Cys, which resulted in the reduced translation of Cys-rich γ-zein (27 kDa) mRNA. However, the development of QPM with high Lys, Trp and vitreous kernel texture depends on the increased expression of 27 kDa γ-zein. Overall, proteome reframing is needed to increase the essential amino acids Lys, Try and Met without compromising kernel texture and agronomic performance.

## 2 Strategies for development of methionine-rich maize lines

### 2.1 Selection of methionine-rich inbred and natural mutant lines

In conventional breeding, the Met concentration in different inbred lines varies greatly. Screening of maize genotypes for high Met percentage followed by suitable breeding program in order to fix this trait along with the better agronomic performance is required to enhance the overall quality. Three cycles of screening and recurrent selection of maize lines can be used to sort the germplasm into different groups based on desired traits. As described in the previous study (Sethi et al., 2021), the temporal expression of δ-zein after pollination can be evaluated to determine the differential pattern of protein accumulation during seed development. It indicates the developmental stage at which maize seeds have maximum Met content that can be used as selection criteria for screening of Met-rich lines. The utilization of grains at that particular developmental stage having maximum Met may help to make the availability of more amount of Met to animals and humans (Sethi et al., 2021). According to earlier reports, the B101 is characterized as a Met-rich line. The parental lines of “Vivek Hybrid 9” (CM145 and CM212) were changed to QPM version through transfer of the *o2* gene with the help of MAS and phenotypic screening for endosperm modifications. It’s QPM version was named “Vivek QPM 9” and released in India during 2008. There was significant improvement of Met by 3.4% in the “Vivek QPM 9” hybrid compared to its normal version “Vivek Hybrid 9” (Gupta et al., 2009; Gupta et al., 2013).

Natural mutants with high Met concentration, such as floury-2 (*fl2*), were identified about 55 years ago (Nelson et al., 1965). The *fl2* encodes unusual α-zeins leading to aberrant protein body formation and an opaque phenotype (Lending and Larkins, 1992). Thereafter, a maize germ line BSSS53 having higher Met content was discovered, as evidenced by higher expression of Met-rich storage protein-producing genes (δ-zein) (Phillips and Mcclure, 1985). Mutations in the genes for *aspartokinase1* (*ask1*) and *aspartokinase2* (*ask2*), encoding enzymes free from feedback inhibition by Lys, have been found in maize (Diedrick et al., 1990). The mutation in these loci resulted in enhanced production of Thr, Lys, Met and isoleucine (Dodson et al., 1990). It has been reported that a maize line Oh54502 with *ask2* mutant showed a higher accumulation of free amino acids in the endosperm (Wang et al., 2007).

### 2.2 Genetic engineering approaches to enhance the methionine content

Since there are a number of control points in and around the metabolic network of Met, conventional breeding may not be able to increase Met levels without compromising yield and quality parameters. As a result, genetic engineering may be a viable option for developing high Met maize lines

#### 2.2.1 Modulating Sulphur-flux toward methionine synthesis

Recent studies suggested that enhancing the accumulation of total sulphur protein and 10 kDa δ-zein without any negative effect on other zeins can be attained by either enhanced S-assimilation in the leaf (Xiang et al., 2018) or decreasing sulfate reduction capacity in the maize kernel (Planta et al., 2017), although sulphur assimilation in kernel cannot be ruled out (Tabe and Droux, 2001). The key enzyme is APR which is not expressed in the kernel. Enhanced expression of two genes *APR* and *SAT* in bundle sheath cells increases the flux through the pathway, resulting in more Met and δ-zeins. However, the accumulation of toxic intermediates in the plants led to stunted growth (Martin et al., 2005), which was the major negative impact on the plant. If this hurdle could be solved, then S-assimilation might potentially enhance the resource for Met accumulation in maize seeds (Wu et al., 2012).

Another enzyme CGS catalyzed the synthesis of Met from Cys. If the activity of this CGS enzyme in maize lines can be increased, more Cys can be synthesized, diverting surplus Cys to Met production and, as a result, higher levels of Met-rich storage proteins in maize kernels can be reached. Amir and Tabe (2006) reported that highly expressive gene of *CGS* from *Arabidopsisthaliana* was transferred into transgenic tobacco (*Nicotiana tabacum*) and alfalfa (*Medicago sativa*) which leads to the accumulation of free Met content in leaves but does not show any effect on the storage protein-bound Met. But, opposite results were attained in transgenic tobacco seeds which express a truncated version of the *Arabidopsis cystathionine-γ-synthase* (*AtCGS*) gene. The feedback-insensitive form of CGS enzyme produces less soluble Met but more bound Met (Matityahu et al., 2013). Both soluble and bound Met was produced in higher amount in *Arabidopsis thaliana* (Cohen et al., 2014) and soybean (Cohen and Amir, 2017) seeds with the same *AtCGS* construct with legume promoter. The feed-back insensitive CGS mutants accumulated Met and performed better under oxidative stress (Cohen et al., 2014). The role of carbon and nitrogenous substrates for Met content has recently been observed, where *aspartokinase* for carbon flow and *CGS* for nitrogen flow were modified with respect to their feedback sensitivity. The binary vector under control of suitable promoters has been transformed into tobacco seeds and leaves resulting in a 2 and 170-fold increase of protein-bound Met, respectively (Hacham et al., 2008; Matityahu et al., 2013). The HMT pulling of accumulated SMM in leaves of *Arabidopsis thaliana* plants with suppressed *CGS* through RNA interference led to 33% increase in seed Met (Cohen et al., 2017). The over-expression of truncated *CGS* in vegetative tissues of rice increased the activity but did not improve the Met content, which once again highlighted the incompetence of rice leaves to throw excess Met to the seed (Whitcomb et al., 2018).

**Figure 3:**
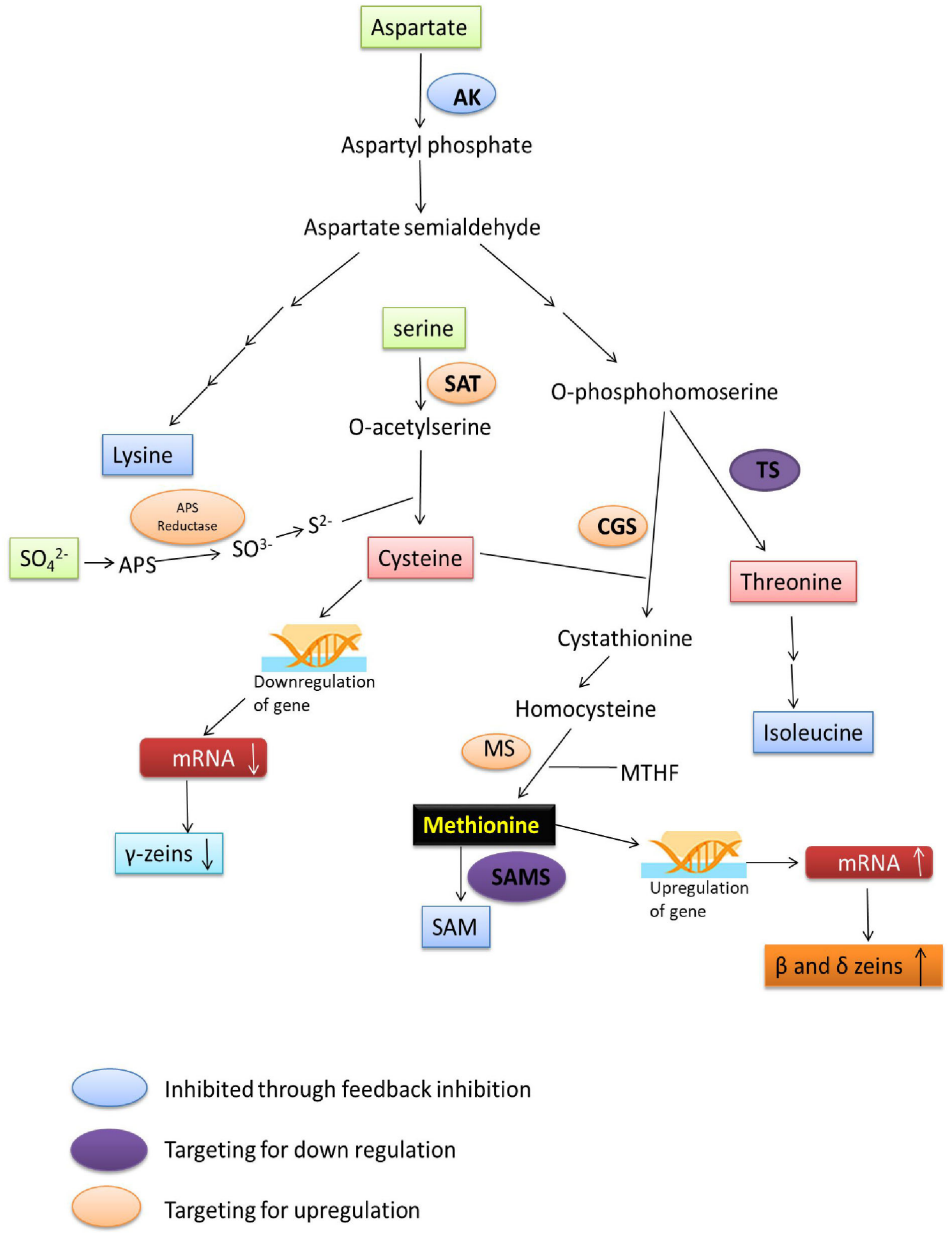
Flow diagram representing target key enzymes for genetic engineering to enhance Met expression. AK, aspartate kinase; SAT, serine acetyl transferase; APS Reductase, adenosine 5-adenylylsulfate Reductase; APS, adenosine 5-adenylylsulfate; CGS, cystathionine-γ-synthase; TS, threonine synthase; MS, methionine synthase; SAMS, S-adenosyl methionine synthase.

In maize CGS, APR and SAT enzymes are the major key regulating enzymes for sulphur flux towards Met synthesis, so up-regulation of these enzymes will result in the enhanced sulphur flow, which finally affects the Met accumulation (**Figure 3**).

#### 2.2.2 Enhancing the sulphur assimilation

The level of Met and zein fractions can be modulated by developing different mutant lines with altered enzymatic activities of sulphur assimilatory or Met biosynthesis pathways. To circumvent many control points, the bacterial *APR* homologs may be excellent candidates to develop Met rich transgenic lines. Under limiting sulphur amino acids (SAA) availability, the B101 exemplified the utmost natural threshold of Met accumulation in maize grain. This threshold apparently can be overcome with increased sulphur-reduction and assimilation during photosynthesis. The role of non-seed tissues in producing and transporting Met to the seed has been studied through genetic engineering. The transgenic maize line developed by using *AtSAT1* gene from *Arabidopsis thaliana* under the control of leaf bundle sheath cell-specific promoter *rbcS1* (Rubisco small subunit 1) exhibited 12-fold higher activity of SAT enzyme and led to higher sulphur assimilation (Xiang et al., 2018). Thus, 4-fold higher amount of soluble Met and Cys in maize lines without any negative impact on maize plants has been observed. Higher Met resulted due to a higher level of 10 kDa δ-zein storage protein, representing 1.40-fold higher protein-incorporated Met in maize kernel (Xiang et al., 2018). Similarly, constitutive over-expression of the *Escherichia coli SAT* gene (*EcSAT*) in rice resulted in a 40% and 4.8-fold increase in soluble and protein-bound Met, respectively (Nguyen et al., 2012). A 17% increase in the bound-Met and 74% increase in Cys of soybean seeds has been observed when *O-acetyl serine (thiol) lyase* gene (*OAS-tL*) was overexpressed (Kim et al., 2012). This finding again confirms the threshold limit of Met accumulation under the presence of pooled Cys. Recently it is reported that the highest Met containing line was developed by stacking the two transgene (*pRbcs : AtSAT1-pRbcs : EcPAPR*), which resulted in 2.24 fold increase of Met in maize (Xiang et al., 2022). Hence, engineering the *SAT* gene of maize for enhanced expression results in higher sulphur assimilation, which positively influences the Met accumulation in the seed (**Figure 3**).

#### 2.2.3 Modulating feedback inhibition

Genetic engineering gives the opportunity to make carbon or sulphur flow-biosynthetic enzymes resistant/insensitive to feedback inhibition, allowing it to produce more enzymes even when feedback inhibitors are present. The chimeric genes producing the Met-rich *2S* albumin of Brazil nuts (*Bertholletia excelsa*) and bacterial feedback insensitive *aspartate kinase* gene have been inserted into narbon bean (*Vicia narbonensis*) (Saalbach et al., 1995; Demidov et al., 2003) and rapeseed (*Brassica napus*) (Altenbach et al., 1992) to improve the amino acid balance of seeds. In maize identifying the different isoforms of the AK insensitive to feedback inhibition and enhanced expression of genes of sulphur assimilation, Met biosynthesis and AK pathway, such as *SAT*, *APR*, *AK*, *CGS* and *MS* could be the potential way to enhance Met accumulation (**Figure 3**).

#### 2.2.4 Overexpression of seed storage proteins

Another approach to enhance amino acid levels is by replacing a storage protein that is lacking in an amino acid with a protein that is enriched with particular amino acid. The sink capacity could be enhanced through the expression of genes encoding Met-rich protein content in the seed. Enhanced expression of Met-rich maize proteins probably led to lower expression of γ- and β-zeins genes (Wu and Messing, 2010). Silencing of genes of γ- and β-zeins proteins showed that these were the main sink of Cys amino acid which act as a donor of sulphur for Met synthesis (Wu et al., 2012).

The higher Met level could be possible at the salvage of other sulphur-containing metabolites and/or enzymes. The reduced synthesis of Cys-containing proteins leads to increased sulphur flux to other amino acids such as Met, resulting in higher δ-zein proteins in the seed (Wu et al., 2012; Wu and Messing, 2014). In the condition of suppression of endogenous sulphur-poor proteins and expression of γ-zeins, available sulphur plays a significant role in the accumulation of Met-rich storage protein (Kim et al., 2014). The expression of sulphur-rich proteins in growing maize seeds was accompanied by a decrease in endogenous sulphur-rich proteins. This suggests that the sulphur shortage triggered the sulphur-protein reallocation (Lai and Messing, 2002; Hagan et al., 2003; Chiaiese et al., 2004). In maize, the seeds having less γ-zeins proein, accumulate 10-56% more Met (Taylor et al., 2008; Yin et al., 2011; Newell et al., 2014). Surprisingly, RNAi-induced gene silencing for genes of β- and γ-zeins proteins has no impact on Met content. Since, Met receives its sulphur mostly from Cys, it appears that the ratio of these two amino acids influences the protein synthesis, and their protein-bound content is regulated by translational altitude (Wu et al., 2012). Recent study showed that the expression of Met-rich maize gene encoding for β-zein protein into soybean under the control of legumin B4 promoter or the CaMV 35S promoter gives the high Met containing transgenic soybean seeds, in which the Met content is significantly increased by up to approximately 15% (Guo et al., 2020). Enhancing expression of Met rich maize delta zein, including 10 kDa δ-zein which is associated with the *dzs10* gene (Zm00001eb382030) located in chr9:47915348-47917942bp and 18 kDa δ-zein associated with the *dzs18* gene (Zm00001eb281380) located in chr6:125301025-125303844bp (MaizeGDB, https://www.maizegdb.org/data_center/map) are key targets to enhance the Met percentage in maize.

In conclusion, targeting the maize protein with a lower Met percentage such as α, β and γ-zeins for its inhibition, or enhancement of protein rich in Met (10 and 18 kDa δ-zeins) will be the potential way for enhancing the protein-bound Met (**Figure 3**).

#### 2.2.5 Overexpression of methionine biosynthesis-related enzymes

Studies revealed that transgenic tobacco seeds expressing bacterial insensitive *aspartokinase* under the seed-specific promoter at the final stage of seed development produce 6.5% more bound Met (Karchi et al., 1993) whereas, overexpression in vegetative tissues led to no significant change in seed Met (Hacham et al., 2008). The latter study suggests that the higher level of Met in leaves does not push the excess into developing seeds.

The enzyme methionine gamma lyase (MGL) is present abundantly in the cytoplasm of all the plant’s organs except seeds which converts Met into methanethiol, ammonia and α-ketobutyrate. It has been reported that this enzyme had a higher k_m_ value for the Met, indicating that the enzyme performed its function when there is higher Met accumulation in the cell (Huang et al., 2014). The higher accumulation of Met and SMM content has been observed in the mutants of *Arabidopsis thaliana* lacking MGL. Over-expression of the *AtCGS* in potato tubers, along with the RNAi : MGL construct, resulted in a 2.2-fold increase in Met (soluble form) accumulation in comparison to the wild-type (Kumar and Jander, 2017). It has also been observed that the MGL enzyme performs its activity under certain conditions such as sulphur starvation, and leads to the degradation of Met (Ufaz and Galili, 2008). The reported studies suggest that the genes of aspartokinase, CGS and MGL enzymes could be targeted to increase the Met percentage *via* genetic engineering techniques.

#### 2.2.6 Gene silencing to enhance methionine

Using a transgenic approach, the levels of protein-bound Met could be enhanced by approximately 30–97% in comparison to their non-transgenic form (Altenbach et al., 1992; Molvig et al., 1997; Lai and Messing, 2002; Lee et al., 2003; Newell et al., 2014). The transgenic *Arabidopsis thaliana* (*mto2*, Methionine over-accumulator) and potato plants with deficient TS enzyme activity produced higher free Met content in leaves (Bartlem et al., 2000; Zeh et al., 2001). The silencing of miRNA reduced the expression of *TS* gene which may lead to the availability of O-homoserine for Met synthesis resulting in enhancement of the Met content of maize endosperm. This has been explained in a way that the enhanced Thr content inhibits the AK enzyme thus inhibiting the synthesis of Met, Lys and Ile. So diverting O-homoserine away from Thr biosynthesis may also prevent the inhibitory effect of Thr. Additionally, accumulation of SAM stimulates the activity of TS enzyme, which inhibits the Met synthesis (Hacham et al., 2002). The Inhibition of the activity of the SAM synthase enzyme may result in higher Met content in maize lines. It has been reported that, the *Arabidopsis thaliana* mutant *mto3* with lower expression of *SAM synthase* gene, resulted in up to 200 folds more Met content. These mutant lines did not show any visible growth difference from the wild type, except for a slight delay in germination (Shen et al., 2002).

Transgenic maize PE5 (contains the expression cassettes PepC-EcPAPR) with *Escherichia coli* gene *3′-phosphoadenosine-5′-phosphosulfate reductase* (*EcPAPR*) under the control of leaf-specific promoter, down-regulates the endogenous *APR* and increases the expression of gene coding for Cys-rich non-zein proteins. These transgenic maize lines produced 57.6% more kernel-bound Met than the best Met-rich maize inbred line B101 (Planta et al., 2017). Transgenic PE5 (maternal) even backcrossed twice to B101 prior to being crossed with α, β, γ, γ/α or γ/β- zein RNAi lines (paternal) produced a higher amount of Met. The loss of β- and γ-zeins, as compared to decreased α-zeins, increased the δ-zeins and produced vitreous kernels only in presence of PE5 i.e. PEF:γ (Wu et al., 2012; Planta et al., 2017). However, some decreases in 27 kDa γ-zeins were attributed to gene segregation (Segal et al., 2003) or gene silencing (Huang et al., 2004). Reduction in both β- and γ-zeins mobilizes even more protein sulphur to the 10 kDa δ-zein than the loss of either β- or γ-zeins individually. δ-zein (10 kDa) seems to be the most responsive to enhanced assimilative sulphate reduction followed by the 15 kDa β-, 16 kDa γ-, and 27 kDa γ-zein, respectively. This order also follows the number of sulphur amino acids in these zeins. Therefore, it seems that the higher the SAA residues of the zeins, the more responsive it would be to increased sulphur supply (Planta et al., 2017).

In maize, targeted inhibition of TS and SAM synthase enzymes will be beneficial to enhance the Met accumulation. Silencing the expression of sulphur-deficient maize proteins may result in the accumulation of sulphur-rich proteins to balance the overall protein percentage and that can be a better approach for developing Met-rich maize germplasm (**Figure 3**).

#### 2.2.7 Modifying gene codons or promoters

By modifying the genes encoding proteins with relatively higher Met in such a way that their encoding regions contain more codons for Met, or engineering the promoter region of those genes to enhance their expression in turn leads to higher accumulation of δ-zein protein in maize endosperm during seed development. This strategy mainly encountered problems of protein instability and relatively small improvement (Krishnan, 2005; Beauregard and Hefford, 2006; Wenefrida et al., 2009). A gene coding for 11 kDa δ-zein protein from maize germ line W23a1 was isolated and introduced into the soya bean plants. These transgenic lines showed 1.5 to 1.7% higher Met content than the non-transgenic lines (Kim and Krishnan, 2004). Till now no modified maize storage proteins are available. According to a study, the expression of the 18 kDa δ-zein protein encoding gene is minimal in most maize plants. Alteration in UTRs of the 18 kDa δ-zein encoding gene may lead to the higher accumulation of Met-rich proteins due to increased gene expression. Recently Planta et al. (2017) used this strategy with *E. coli* PAPS (3’-phosphoadenosine-5’-phosphosulfate) reductase (PPAR) and maize δ-zein and improved the Met content for 58% as compared to control without apparent proteome rebalancing. Thus, targeting the δ-zein protein or δ-zein encoding gene is important for Met regulation. The existence of alternative routes for Met synthesis through PPAR provides flexibility in metabolic engineering in maize.

#### 2.2.8 Expression of synthetic genes

This strategy can be used to generate transgenic higher Met-maize lines expressing codon-optimized synthetic genes having kernel-specific promoters. It will help to transform Met, to change an imbalanced composition into a balanced one, and for that non-conserved regions of proteins must be replaced with a sequence high in Met. In sweet potato (*lpomoea batatas*) and soybean, seed-specific expression of synthetic genes (*MB-16* for soybean) resulted in seed protein with 13-16 percent (higher) Met residues (Slater et al., 2008; Zhang et al., 2014). De Clercq et al. (1990) constructed the Met-rich *AT2S1* gene by replacing the poorly conserved region with Met-rich sequences of *2S* albumin gene 1 of *Arabidopsis thaliana* and the insertion of that construct in *Arabidopsis thaliana*, Brazil nut and tobacco resulted into Met-enriched 2S albumins in all of them. The new chimeric gene *zeolin* was designed to encode Met-rich proteins through fusion of maize γ-zein with bean storage protein phaseolin (Mainieri et al., 2004). This strategy has scope to form stable chimeric proteins with the required content of essential amino acids. The identification of non-conserved/non-functional regions from the maize genome and replacement of them with Met-rich sequence could be an alternative option to generate Met-rich transgenic maize lines.

### 2.3 Targeting identified candidate genes associated with methionine *via* genetic engineering/gene editing

Currently, gene editing is a potential method for altering a particular region of interest. In this work, a comprehensive list of genes (source MaizeGDB) associated with Met production has been prepared (**Table 3**) which can be used to enhance its level via various genetic engineering approaches. The 25 loci (S No. 36-60 in **Table 3**) were found to be associated with Met content using GWAS experiments across two environments in the previous study (Deng et al., 2017) which can be used for molecular screening of maize germplasm for higher Met content. Some of the genes for overexpression *via* genetic engineering are also listed (**Table 4**). It is well known that for gene editing Met-rich lines there is a need to target the negative regulators of Met production. Therefore, the list has been prepared for prospective candidate genes which can be knockout to develop transgene-free Met-rich genome-edited (GEd) maize lines (**Table 4**) *via* CRISPR-Cas9-based gene-editing technique.

**Table 3:** Details of candidate genes associated with methionine metabolism, which codes for enzymes, transcription factors, signaling and transporter proteins.

S. No.	Candidate gene	Metabolite	Chr	Annotation	Position (bp)	Reference
1.	GRMZM2G024686	Met	1	Aspartate kinase, conserved site	298313666-298414802	Wen et al., 2014
2.	GRMZM2G406746	Met	7	Pentatricopeptide repeat	9769078-9872377	
3.	GRMZM2G110145	Met	10	Cellulose synthase	77271453-77305285	
4.	GRMZM2G128319	Met	10	Protein kinase-like	113791164-113887667	
5.	GRMZM2G121275	Met	2	Major intrinsic protein	19780391-20248092	
6.	GRMZM2G121223	Met	2	Lipid-binding START	19780391-20248092	
7.	GRMZM2G702954	Met	2	Unknown	19780391-20248092	
8.	GRMZM2G101290	Met	2	Glucose/ribitol dehydrogenase	19780391-20248092	
9.	GRMZM2G101181	Met	2	Protein of unknown functions DUF246, plant	19780391-20248092	
10.	GRMZM2G078648	Met	2	Zinc finger, FYVE/PHD-type	19780391-20248092	
11.	GRMZM2G172936	Met	2	Pathogenesis-related transcriptional factor and ERF, DNA-binding	19780391-20248092	
12.	GRMZM2G411639	Met	2	Class I peptide chain release factor	19780391-20248092	
13.	GRMZM2G028969	Met	2	Pathogenesis-related transcriptional factor and ERF, DNA-binding	19780391-20248092	
14.	GRMZM2G325513	Met	2	Antifreeze protein, type I	19780391-20248092	
15.	AC207188.3_FG002	Met	2	Unknown	19780391-20248092	
16.	GRMZM2G105137	Met	2	SANT, DNA-binding	19780391-20248092	
17.	GRMZM2G139458	Met	2	Unknown	19780391-20248092	
18.	GRMZM2G139463	Met	2	Peptidase T2, asparaginase 2	19780391-20248092	
19.	GRMZM2G024389	Met	1	Unknown	298313666-298414802	
20.	GRMZM2G024267	Met	1	Unknown	298313666-298414802	
21.	GRMZM2G024374	Met	1	Kinesin, motor region	298313666-298414802	
22.	GRMZM2G023242	Met	1	Nascent polypeptide-associated complex NAC	298313666-298414802	
23.	GRMZM2G022269	Met	1	Protein synthesis factor, GTP-binding	298313666-298414802	
24.	GRMZM2G022248	Met	1	Glutaredoxin-related protein	298313666-298414802	
25.	GRMZM2G420772	Met	7	Plant disease resistance response protein	1262063-1309782	
26.	GRMZM2G420743	Met	7	Plant disease resistance response protein	1262063-1309782	
27.	GRMZM2G420758	Met	7	Unknown	1262063-1309782	
28.	GRMZM2G420733	Met	7	Plant disease resistance response protein	1262063-1309782	
29.	GRMZM2G120652	Met	7	Vitamin B6 biosynthesis protein	1262063-1309782	
30.	GRMZM2G120575	Met	7	Protein of unknown function DUF1665	1262063-1309782	
31.	GRMZM2G120574	Met	7	Tyrosine protein kinase	1262063-1309782	
32.	GRMZM2G120572	Met	7	Unknown	1262063-1309782	
33.	GRMZM2G555108	Met	7	WD40 repeat	1262063-1309782	
34.	GRMZM2G120563	Met	7	C2 calcium/lipid-binding region, CaLB	1262063-1309782	
35.	GRMZM2G109627	Met	8	No apical meristem (NAM) protein	1262063-1309782	
36.	GRMZM2G095631 (chr4.S_46137424)	Met	4	Unknown	46137424	Deng et al., 2017
37.	GRMZM2G160541 (chr4.S_143379704)	Met	4	Phenylalanine ammonia-lyase	143379704	
38.	GRMZM2G139412 (chr5.S_151841984)	Met	5	Shikimate kinase	151841984	
39.	GRMZM2G362298 (chr6.S_74144428)	Met	6	Acyl-transferase family protein	74144428	
40.	GRMZM2G351239 (chr1.S_190429440)	Met/AAT	1	Unknown	190429440	
41.	GRMZM2G159145 (chr3.S_13532873)	Met/AAT	3	Thioredoxin H-type	13532873	
42.	GRMZM2G160541 (chr4.S_143379704)	Met/AAT	4	Phenylalanine ammonia-lyase	143379704	
43.	GRMZM2G065451 (chr4.S_237774932)	Met/AAT	4	Transcription factor family protein	237774932	
44.	GRMZM2G425728 (chr7.S_9707633)	Met/AAT	7	Early light-induced protein	9707633	
45.	GRMZM2G015534 (chr7.S_10695002)	Met/AAT	7	Opaque endosperm2	10695002	
46.	GRMZM2G344911 (chr7.S_88939234)	Met/AAT	7	2,3-dihydro-2,3-dihydroxybenzoate dehydrogenase	88939234	
47.	GRMZM2G133806 (chr8.S_164189020)	Met/AAT	8	Unknown	164189020	
48.	GRMZM2G091819 (chr10.S_16572309)	Met/AAT	10	Disulfide oxidoreducatse	16572309	
49.	GRMZM2G351239 (chr1.S_190429440)	Met/Total	1	Unknown	190429440	
50.	GRMZM2G013283 (chr2.S_50503804)	Met/Total	2	Glutamate-ammonia ligase	50503804	
51.	GRMZM2G158316 (chr4.S_185735097)	Met/Total	4	Unknown	185735097	
52.	GRMZM2G425728 (chr7.S_9707633)	Met/Total	7	Early light-induced protein	9707633	
53.	GRMZM2G045834 (chr7.S_28399595)	Met/Total	7	Unknown	28399595	
54.	GRMZM2G133806 (chr8.S_164189020)	Met/Total	8	Unknown	164189020	
55.	GRMZM2G008226 (chr1.S_218566308)	Met	1	trehalose biosynthetic process	218566308	
56.	GRMZM2G166713 (chr4.S_165967277)	Met	4	methionine-tRNA ligase	165967277	
57.	GRMZM2G178106 (chr9.S_153267892)	Met	9	Beta-galactosidase	153267892	
58.	GRMZM2G175463 (chr9.S_153269528)	Met	9	Unknown	153269528	
59.	GRMZM2G176141 (chr.S_180848700)	Met/AAT	2	Transcription factor	180848700	
60.	GRMZM2G138727 (chr7.S_120199552)	Met/Total	7	Glutelin-2 Precursor (Zein-gamma) (27 kDa zein)	120199552	

*ATT=Aspartate-derived amino acid family related traits, Total= Total amino acids.

**Table 4:** Details of proposed genes to be targeted for the genetic engineering and genome editing for methionine.

S. No.	Candidate gene	Chr	Position (bp)	Annotation	Strategy	Reference
1.	Zm00001eb064530 (*ask1*)	1	305262144-305265827	Aspartate kinase	Knockout	Bartlem et al., 2000; Zeh et al., 2001; Avraham et al., 2005; Kumar and Jander, 2017; Hou et al., 2022; MaizeGDB
2.	Zm00001eb094670 (*ask2*)	2	161226305-161232000	Aspartate kinase		
3.	Zm00001eb392050 (*CGS1*)	1	65940342-659460321	Cystathionine-γ-synthase	Over-expression	
4.	Zm00001eb018300	1	65198840-65205925	Cystathionine-γ-synthase		
5.	Zm00001eb156060	3	212214968-212222772	Threonine synthase	Knockout	
6.	Zm00001eb294790 (*thr1*)	6	174066198-174071347	Threonine synthase		
7.	Zm00001eb284240 (*thr2*)	6	143594078-143599133	Threonine synthase		
8.	Zm00001eb022690 (*thr3*)	1	87258448-87262005	Threonine synthase		
9.	Zm00001eb088230	2	107810019-107813351	Threonine synthase		
10.	Zm00001eb361640 (*sat1*)	8	160277043-160280293	Serine acetyltransferase	Over-expression	
11.	Zm00001eb008110 (*sat2*)	1	24415858-24419122	Serine acetyltransferase		
12.	Zm00001eb293180 (*sat3*)	6	163558296-163561657	Serine acetyltransferase		
13.	Zm00001eb002740 (*sat4*)	1	7638621-7653175	Serine acetyltransferase		
14.	Zm00001eb322440 (*aprl1*)	7	157694136-157699277	PAPS Reductase	Down-regulation	
15.	Zm00001eb105800 (*aprl2*)	2	209131699-209136961	PAPS Reductase		
16.	Zm00001eb194120 (*aprl3*)	4	183478812-183483064	PAPS Reductase		
17.	Zm00001eb255320 (*aprl4*)	5	214156431-214161113	PAPS Reductase		
18.	Zm00001eb042300 (*aprl5*)	1	221287901-221308523	PAPS Reductase		
19.	Zm00001eb410330 (*aprl6*)	10	25080671-25087625	PAPS Reductase		
20.	Zm00001eb137590 (*aprl7*)	3	133490011-133494737	PAPS Reductase		
21.	Zm00001eb061530 (*aprl8*)	1	295632186-295637314	PAPS Reductase		
22.	Zm00001eb212580 (*aprl9*)	5	3625345-3630121	PAPS Reductase		
23.	Zm00001eb177530 (*akh1*)	4	68460416-68515471	Homoserine dehydrogenase	Over-expression	
24.	Zm00001eb097580 (*akh2*)	2	177654848-177677022	Homoserine dehydrogenase		
25.	Zm00001eb382030 (*dzs10*)	9	47915348-47917942	10kDa δ-zein	Over-expression	
26.	Zm00001eb281380 (*dzs18*)	6	125301025-125303844	18kDa δ-zein		

#### 2.3.1 Combinatorial strategy

**Figure 4:**
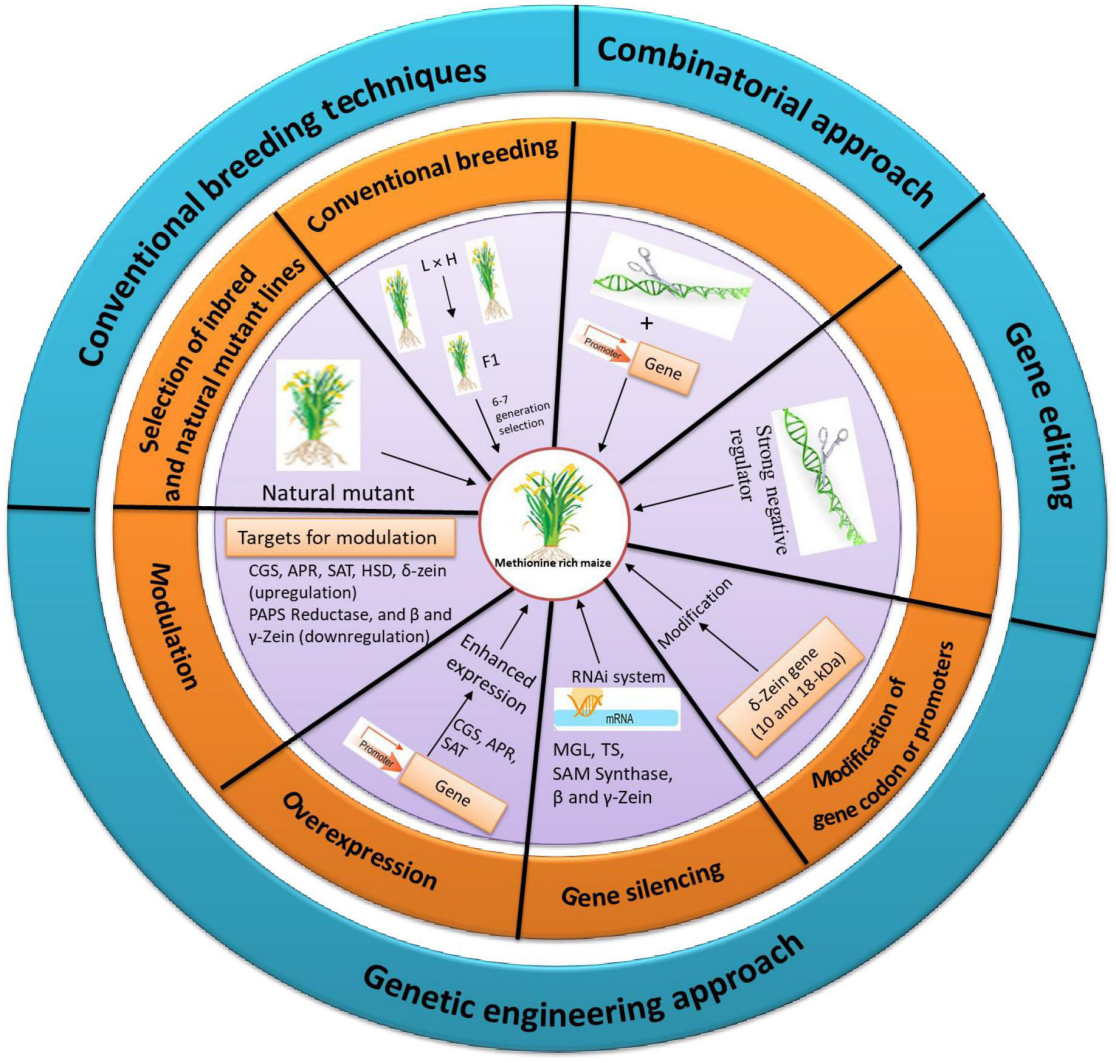
Different approaches and targets for the enhanced accumulation of methionine in maize.

As methionine regulation is a complex process the strategies have to be used in combination to enhance the Met content. This combinatorial strategy works on improved sink and source relationship. In sink capacity, Met enriched albumin protein was added whereas the source was improved through making carbon family enzyme insensitive. Similar combinatorial strategy with insensitive SAT and sunflower albumin in lupin increased the Met content to 2-folds but the dosage effect of SAT was negligible (Tabe et al., 2010). Recently, in soybean, although the introduction of β-zein gene improved total Met content in the seeds, this level was negligible compared to native soybean storage proteins, implying that the inadequate soluble Met is the limiting factor. From these findings it is clear that the Met regulation is a multi-step process, and to up-regulate, a combined strategy needs to be designed for simultaneous increase of the source and sink of the Met metabolism. Various approaches to enhance the Met level in maize seed are represented in **Figure 4**.

## 3 Conclusion and future directions

The enhancement of Met in the seed for balancing the amino acid content is a tricky process. A number of metabolic enzymes, transporters, genes and transcription factors are involved in regulating the biosynthesis and accumulation of Met. Apart from it, temporal separation for Met transporting molecules and spatial separation of sulphur assimilation makes the control of whole biosynthetic pathway a difficult task. The tissue-specific expression of Cys in bundle sheath cells and glutathione in mesophyll cells compartmentalizes the sulphur at the next higher level of cellular hierarchy. Hence, a combination of multiple approaches without compromising the yield is required to be adopted. The genomic regions associated with the trait identified in orthologous cereal crops could be used for identifying hotspot genomic regions in maize for the trait of interest. This review provides trait-related information which can be used further in metaQTL analysis to study the congruency of the identified regions associated with specific traits of interest. The strategies explained in this review will give potential insight into target enzymes and candidate genes, which can be used to enhance Met content *via* genetic engineering or genome-editing approaches. Although these strategies seem to be promising, their effectiveness depends upon the additional studies to define the effect of these manipulations on traits such as seed morphology, seed starch, amino acids, oil content and germination rate. Thus, genetic manipulation had proven successful in increasing the content of Met in seeds. Nevertheless, supplemental information is required to confirm the *in vivo* stability of genetically engineered Met-rich proteins for optimal fortification of seeds. Once nutritionally improved genetically modified (GM)/genetically edited (GEd) genotypes possessing high Met content in their seeds are being developed, there will be an urgent need to analyze the genotype with the best response for superior agronomic traits to perform well under field conditions.

## Author contributions

Conceptualization, DC and VD; writing-original draft preparation, VD; writing-review and editing, BB, VS, MS, AS, CK, MG, SR, RK, and DC; supervision, DC. All authors contributed to the article and approved the submitted version.
